# Testing the Induction of Metritis in Healthy Postpartum Primiparous Cows Challenged with a Cocktail of Bacteria

**DOI:** 10.3390/ani13182852

**Published:** 2023-09-08

**Authors:** Josiane C. C. Silva, Leonardo Bringhenti, Lucas C. Siqueira, Marjory X. Rodrigues, Martin Zinicola, Brianna Pomeroy, Rodrigo C. Bicalho

**Affiliations:** 1 Department of Population Medicine and Diagnostic Sciences, Cornell University, Ithaca, NY 14853, USA; 2 Zoetis Animal Health, Kalamazoo, MI 49007, USA; 3 FERA Diagnostics and Biologicals, College Station, TX 77845, USA

**Keywords:** metritis, challenge, microbiota, primiparous, dairy cow

## Abstract

**Simple Summary:**

Here we tested whether a metritis induction model would induce disease in postpartum primiparous cows. Animals were challenged with an intrauterine infusion of a bacterial cocktail containing 10^3^ or 10^6^ cfu of *Escherichia coli*, *Trueperella pyogenes*, and *Fusobacterium necrophorum*. Both challenged groups were compared with a control group, infused with a sterile saline solution. The incidence of metritis did not differ among groups, but the high-dose group (10^6^ cfu) had a 2.7 times greater hazard of being diagnosed with metritis when compared with the controls and had reduced milk production, which is associated with a numerical reduction in dry matter intake when compared with the other two groups. Although clinical signs of illness were detected in low-dose cows, there were no significant changes in blood concentrations of acute phase proteins, plasma cytokines, or serum metabolites after the challenge. The culture of uterine content revealed that challenging increased the bacterial counts in the vaginal discharge of *Fusobacterium necrophorum*, but not *Escherichia coli* or *Trueperella pyogenes*.

**Abstract:**

Metritis is a postpartum uterine disease with greater incidence in primiparous than in multiparous cows. In primiparous cows, the impact on production and health is lessened, presumably due to a superior immune response. Here, we tested whether an in vivo model of clinical metritis induction developed for postpartum multiparous Holstein cows would produce similar results in primiparous cows. Thirty-six cows were randomly assigned to one of three groups and received intrauterine infusion within 24 h of parturition. The controls were infused with sterile saline; the low-dose group received a bacterial cocktail containing 10^3^ cfu of *Escherichia coli*, *Trueperella pyogenes*, and *Fusobacterium necrophorum*; and the high-dose group were infused with 10^6^ cfu of the same cocktail. Production, health traits, and the vaginal discharge culture were assessed daily, from enrollment until 14 d in milk. Clinical metritis occurred in 64% of high-dose cows, 33% of the controls, and 42% of low-dose cows, with no significant difference of incidence between groups. However, when accounting by time, high-dose cows had a 2.7 times greater hazard of metritis compared with the controls. The bacterial challenge affected milk production and dry matter intake tended to decrease. In the high-dose group, a greater growth *of F. necrophorum* in the selective medium was also observed, suggesting an association with metritis. Therefore, this study suggests intrauterine inoculation with 10^6^ cfu of this bacterial cocktail elicits physical and clinical outcomes consistent with clinical metritis.

## 1. Introduction

The transition period, defined as three weeks before to three weeks after parturition [1], is a critical period for lactating dairy cows. Since postpartum feed intake is not sufficient to meet the nutritional demands of lactation, dairy cows must adapt physiologically. This adaptation includes the uncoupling of the somatotropic axis, increased insulin resistance, and lipolysis [2]. Infectious diseases occur frequently early postpartum, as glucose, an important energy substrate for the immune system, is consumed by the mammary gland for the production of milk [2,3,4,5]. Metritis is one of the most common infectious diseases in postpartum cows, with an incidence of 25–40% in the first week after calving [6].

Metritis is defined via an abnormally enlarged uterus with a fetid, watery red-brown uterine discharge, which is associated or not with signs of systemic illness including dullness, decreased milk production, and signs of toxemia [7]. This multifactorial disease is associated with twins, stillbirth, the delivery of male calves, dystocia, caesarian section, retained placenta, ketosis, and hypocalcemia [7,8,9,10]. Bacteria may enter the uterus by ascending from feces, skin, and the environment when the physical barriers of the uterus are disrupted at calving, or through blood, as some microorganisms present in the infected uterus are not found in the vaginal samples [11,12]. It has been widely described that the microorganisms typically associated with metritis are *Escherichia coli*, *Trueperella pyogenes*, *Fusobacterium necrophorum*, and the bacteria of the genera *Prevotella* and *Bacteroides* [13,14,15]. Virulence factors known to be harmful to the reproductive tract have been described for each of these bacterial species: *Escherichia coli* possesses *FimH*, *Trueperella pyogenes* carries pyolysin, and *Fusobacterium necrophorum* produces leukotoxin.

Metritis is a concern to the dairy industry as it compromises animal welfare, health, and productivity. The average cost associated with a single case of metritis is estimated to be $513, with 95% of cases ranging from $240 to $884 [16,17].

Primiparous have a greater incidence of metritis when compared with multiparous cows, likely due to the need for assistance during calving, increasing the likelihood of bacterial contamination of the uterus [18,19,20]. Both parities, however, have different responses to metritis, as the detrimental effects on milk production, dry matter intake (DMI), and cull rate are more severe in multiparous compared with primiparous cows [21,22]. Authors speculate that this may be due to the less pronounced negative energy balance [23] and the faster immune response [24] observed in primiparous cows.

Here, we tested whether metritis could be induced in primiparous cows using a model validated for multiparous cows [25]. First, lactation cows received intrauterine infusion with a low [10^3^ (colony forming units) cfu] or a high (10^6^ cfu) dose of *Escherichia coli*, *Trueperella pyogenes*, and *Fusobacterium necrophorum* within 24 h after parturition and were compared with the control cows that received an infusion of the sterile saline solution. Clinical, metabolic, and inflammatory responses were measured.

## 2. Materials and Methods

### 2.1. Sample Size Calculation

The incidence of metritis at the commercial farm where the cows were purchased ranges from 8 to 16%; therefore, assuming a type 1 error of 5% and a power of 80%, a sample size of 12 animals per group will create an increase in the metritis incidence to 75% among the challenged and control groups.

### 2.2. Animals, Facilities, and Management

Cows were purchased from a single commercial farm located in Upstate New York (Scipio Center, NY, USA), between June and September 2020. Immediately after parturition, cows that met all the inclusion criteria were challenged at the maternity pen with the assigned bacterial cocktail or the control and transported to the Cornell Teaching Dairy Barn, where they stayed until the end of the study period (15 d in milk).

The commercial farm had approximately 4100 cows in lactation, housed in free-stall barns with manure solids bedding, providing access to *Ad libitum* water and a total mixed ration (TMR), according to production. The Cornell Teaching Dairy Farm is located in Ithaca, NY, with a capacity to house approximately 200 lactating cows in free-stall barns, with free access to water and TMR, fan ventilation, and sawdust bedding. The tie stall barn, where the study was conducted, has space for eight cows, with wood shaving bedding and fan ventilation. Cows had individual access to Ad libitum water and feed bins that allowed the measurement of the daily feed intake.

Milking was performed twice daily, and the milk weights were recorded. Feed was offered daily during the morning (between 07:00 and 08:00 h) at 110% of the expected daily consumption. Feed intake was determined via the difference in weights of the offered and refused feed; dry matter was measured daily using aliquots of TMR that were dried at 55 °C for 48 h. Daily feed intake and dry matter were used for the calculation of DMI. Diet formulation and chemical composition were obtained from the reports received from the nutritionist responsible for the farm and presented in Tables S1 and S2.

### 2.3. Experimental Design

The study had a randomized complete block design with a one-way treatment structure, and the block factor was the calving order (order of enrollment). The treatment randomization was generated using the SAS statistical package (version 9.4; SAS/STAT, SAS Institute Inc.; Cary, NC, USA) that uses a random number generator function created by the Biometrics Representative (BMR) from Zoetis Animal Health (Kalamazoo, MI, USA). One study member was assigned for the challenge administration, and two other study members were blinded to treatments and responsible for sample collection and metritis diagnosis.

At any sign of parturition, cows were moved to the maternity pen and assisted by trained farm personnel helping as needed. After parturition, the date and h of calving, cow and calf ID, calf sex and weight, ease of calving score, and person present at parturition were recorded and provided to the research group at the time of enrollment. Only healthy primiparous cows that met the inclusion criteria (gestation length between 270 and 285 d, giving birth to a single offspring without assistance, not presenting placenta at the time of the challenge, and not receiving antibiotic treatment at least 30 d before calving) were enrolled in the study. Cows were considered healthy if not presenting dehydration, empty rumen, recumbence, dullness, depression, lameness, respiratory diseases, displaced abomasum, mastitis, and/or vaginal tear at the time of enrollment. Post enrollment exclusion happened if the animal presented recumbence due to trauma after calving (e.g., bone fracture or nerve paralysis, toxic diseases not related with metritis, and/or milk fever), developed serious diseases during the postpartum period (e.g., hardware disease, displaced abomasum, respiratory diseases, and/or clinical hypocalcemia), was found injured or dead before completion of the study, received systemic antibiotics for concurrent diseases (not including mastitis treated with intramammary antibiotics), or presented a retained placenta (not visible at the time of enrollment).

A total of 36 primiparous cows were selected and evenly assigned to one of the three treatments groups. The control group received an intrauterine infusion with 120 mL of the sterile saline solution (TEKnova; Hollister, CA, USA); the low-dose group received 120 mL of an inoculum containing 10^3^ cfu of *E. coli*, *T. pyogenes*, and *F. necrophorum*; and the high-dose group received 120 mL of an inoculum containing 10^6^ cfu of *E. coli*, *T. pyogenes*, and *F. necrophorum*. One cow enrolled in the high-dose group was euthanized on study d 4 due to severe pneumonia. Therefore, 35 cows concluded the follow-up period of 14 d.

### 2.4. Inoculum Preparation

An inoculum containing *E. coli*, *T. pyogenes*, and *F. necrophorum* was used for the bacterial challenge. The strains were isolated from the uterus of cows diagnosed with metritis, which belong to our bacterial collection (Bicalho Laboratory at Cornell University; Ithaca, NY, USA) and were selected according to the presence of virulence genes [26,27,28,29].

*E. coli* were cultured under aerobic conditions on Luria–Bertani (LB) broth (Sigma-Aldrich; St. Louis, MO, USA), with agitation (3× *g*) at 37 °C for 24 h. Following incubation, the culture was centrifuged to harvest the cells (2500× *g* for 10 min, room temperature), and the LB broth was supplemented with 25% *v*/*v* of glycerol, which was used to resuspend the cell pellet to a final count of 10^9^ cfu/mL. The final suspension was aliquoted in 2 mL cryogenic vials, flash frozen in liquid nitrogen, and stored at −80 °C.

An incubator supplemented with 5% CO_2_ was used to culture *T. pyogenes* in a VersaTREK REDOX 1 media broth (Thermo Fisher Scientific; Waltham, MA, USA) for 48 h at 37 °C. After incubation, the cells were harvested via centrifugation in room temperature (2429× *g* for 10 min), and the pellet was resuspended to a final count of 10^9^ cfu/mL in a microbial freeze-drying buffer (OPS Diagnostics; Lebanon, NJ, USA) and aliquoted in 2 mL cryogenic vials that were lyophilized in the Advantage Pro lyophilizer (SP Scientific; Warminster, PA, USA) according to manufacturer instructions. The vials were then sealed and stored at 4 °C.

The BACTRON 300 anaerobic incubator (Sheldon Manufacturing Inc.; Cornelius, OR, USA) was used to culture *F. necrophorum* in a VersaTREK REDOX 2 Media broth (Thermo Fisher Scientific), for 48 h at 37 °C. Following incubation, the culture was centrifuged in 2429× *g* for 10 min to harvest the cells. The cell pellet was resuspended to a final count of 10^9^ cfu/mL using a VersaTREK REDOX 2 (Thermo Fisher Scientific) media broth supplemented with 25% *v*/*v* of glycerol, aliquoted in 2 mL cryogenic vials, flash frozen in liquid nitrogen, and stored at −80 °C.

Two to four hours before use in the field, the stocks were transferred to 40 mL bottles containing the specific transport media. The Versa TREK REDOX 1 media was used to transport *E. coli* and *T. pyogenes*, while the Versa TREK REDOX 2 Media was used to transport *F. necrophorum*.

### 2.5. Evaluation of Bacterial Culture Purity

The bacterial stocks of each strain were submitted to the quality control assessments between June and September 2020, to confirm the purity and bacterial count (cfu/mL). DNA was extracted from the stocks, followed by amplification of the 16S rRNA gene and Sanger sequencing (Cornell University Biotechnology Institute) in order to confirm the bacterial species as previously described [30].

Bacterial counts were performed using the technique of Agar droplets with some modifications. Briefly, serial dilutions were prepared (10^−1^ to 10^−8^) using sterile 1× PBS (pH 7.4) for each bacterial strain. Next, each quadrant of agar plates was inoculated with three drops of each dilution and incubated according to the bacterium characteristic. After this, the bacterial growth was counted and the number of cfu determined.

*E. coli* was cultured in blood agar and mastitis GN plates (CHROMagar; Paris, France) under aerobic conditions at 37 °C for 24 h. *F. necrophorum* was cultured in blood agar and LKV agar plates (Laked Blood with Kanamycin and Vancomycin; Anaerobe Systems; Morgan Hill, CA, USA) for 48 h at 37 °C under anaerobic conditions. Lastly, *T. pyogenes* was cultured in blood agar plates in a 5% CO_2_ supplemented incubator for 48 h at 37 °C.

### 2.6. In Vivo Experimental Challenge

At the maternity pen, cows were restrained in a headlock, and the perineal area and vulva were cleaned with a paper towel followed by disinfection with 70% ethanol. A sterile gilt foam tip catheter (QC Supply; Schuyler, NE, USA) was attached to a 60 mL syringe (Air-tite products Co., Inc.; Virginia Beach, VA, USA) and used for inoculum administration. The catheter was introduced into the cranial vagina and manipulated via the cervix to access the uterine lumen. One syringe was used for administration of each bacterial culture (40 mL of volume) into the uterus. To assure that all the challenge was infused into the uterus, the catheter was flushed with the sterile saline solution (10 mL). No adverse effects of the challenge administration, other than metritis development, were expected as the strains used are found in healthy and metritic cows.

### 2.7. Animal Sampling

Prior to the challenge administration, at the moment of enrollment, blood samples and vaginal swabs were collected in order to determine the baseline for all the parameters measured, where the data is described as d 0. The day after enrollment, samples were collected during the morning until the end of the study period (d 14).

Blood samples were collected during the morning from coccygeal vessels before administration of the challenge and daily from study d 1 to 14. A 3 mL vacutainer K2 EDTA tube (BD Vacutainer; Franklin Lakes, NJ, USA) was used to perform genomic testing (CLARIFIDE, Zoetis Animal Health; Kalamazoo, MI, USA) and a complete blood cell count (CBC; Heska–Hemature^TM^; Loveland, CO, USA). After processing the CBC, the blood samples were centrifuged at 2000× *g* for 15 min at room temperature for plasma separation and frozen at −80 °C. Frozen plasma samples were sent to a Zoetis Research and Development facility (Kalamazoo, MI, USA) for measurement of the following acute phase proteins using commercial ELISA kits: lipopolysaccharide binding protein (LBP) (HycultBiotech; Wayne, PA, USA), serum amyloid A (SAA) (Life Diagnostics, Abu Dhabi, United Arab Emirates), and haptoglobin (Life Diagnostics). Proinflammatory cytokines such as tumor necrosis factor alpha (TNFα), interleukin (IL)-6, IL-2, IL-8/CXCL8, and IL-10 were also measured in plasma levels using two custom U-Plex assays [Meso Scale Diagnostics (MSD), LLC; Rockville, MD, USA] developed for bovine. Analysis of TNFα, IL-2, IL-6, and IL-10 were performed in a 4-Plex assay format, while a 1-Plex format was used for bovine IL-8/CXCL8. Briefly, biotinylated antibodies were diluted using MSD Diluent 100 to a final concentration of 10 µg/mL and individually linked to specific MSD Linkers following the manufacturer’s recommendations. The linked antibodies were diluted in MSD Stop Solution: 4-Plex: TNFα (1:10), IL-2 (1:10), IL-6 (1:10), and IL-10 (1:10); 1-Plex: IL-8/CXCL8 (1:10). The 4-Plex linked antibodies were combined into one antibody capture solution and the 1-Plex linked antibody was prepared as a separate antibody capture solution. The capture antibody solution (50 μL/well) was added to the respective U-Plex assay plates and incubated for a 1 h shaking at room temperature. After incubation, the plates were washed three times using PBS added with 0.05% Tween-20. The samples for the 4-Plex measurements were not further diluted, while the samples for the 1-Plex measurement were diluted to 1:100 in SeaBlock (Thermo Fisher), where the duplicates of the samples were added to each plate. Plates were incubated at room temperature for an interval of 1 to 1.5 h shaking with the samples and standards. Following incubation, the plates were washed five times using PBS added with 0.05% Tween-20. A total of 30 µL/well of detection antibody solutions conjugated with MSD SULFO-TAG were added, followed by a 1 h incubation at room temperature by shaking. The plates were then washed one time with detection antibodies, followed by three times washed using PBS added with 0.05% Tween-20. Lastly, 150 µL/well of 2 x MSD Read Buffer was added to the plates and the reading was performed immediately on a MESO SECTOR S 600MM instrument. The upper limit of detection (ULD), lower limit of detection (LLD), and the intra-assay coefficient of variation (CV) for each of the cytokines measured were IL-8 (ULD: 200,000; LLD: 48.83; CV: 6.07); IL-10 (ULD: 80,000; LLD: 19.53, CV: 1.93); IL-2 (ULD: 80,000; LLD: 19.53; CV: 2.27); IL-6 (ULD: 80,000; LLD: 19.53, CV: 2.12); and TNFα (ULD: 80,000; LLD: 19.53; CV: 2.32). To enable downstream analysis, any value that felt between zero and the LLD was replaced by ½ of the LLD.

A 10 mL vacutainer tube with spray-coated silica (BD Vacutainer; Franklin Lakes, NJ, USA) was used for serum separation. Tubes were centrifuged at 2000× *g* for 15 min at room temperature and frozen at −80 °C. A serum concentration of calcium, fatty acids (FA), BHB, total protein, bovine serum albumin, glucose, alkaline phosphatase (ALP), gamma glutamyl transferase (GGT), aspartate aminotransferase (AST), alanine aminotransferase (ALT), and lactate were measured using an automated clinical chemistry analyzer (Daytona, Randox Laboratories Ltd.; Kerneysville, WV, USA), using reagents provided by the same company.

From enrollment to the end of the study period (study d 14), a daily measurement of rectal temperature (RT) was performed. Fever was defined as RT ≥ 39.5 °C for at least one day during the study period. At enrollment and on study d 14, the body weight (BW) and body condition score (BCS) [31] were performed for the estimation of losses. The BW scale malfunctioned at enrollment and at the end of the study for some cows, causing data losses. Therefore, the BW results are based on a reduced number of cows per group. Statistical analysis of BW was thus performed on seven cows enrolled in the control group, eight in the low-dose group, and eight cows in the high-dose group.

### 2.8. Vaginal Discharge Sample Collection

Vaginal discharge was evaluated daily (Metricheck, SimcroTech; Hamilton, New Zealand). The Metricheck device was kept immersed in a recipient containing ammonium persulfate when not in use. Briefly, the perineal area and vulva were cleaned with a paper towel and disinfected with 70% ethanol, and the Metricheck device was rinsed with sterile distilled water, introduced in the cranial extent of the vagina, and then retracted caudally, bringing the material adhered to its silicone hemisphere. Right after use, the Metricheck was cleaned with a paper towel in order to remove any uterine content remaining, rinsed with sterile distilled water, and placed into the solution containing ammonium persulfate until use in the next cow. The same Metricheck device was used for the collection of uterine content in all cows independently of the group assignment.

Culture dependent (plate counting) and culture independent (16S rRNA sequencing) methods were used to determine the bacterial load in the vaginal discharge retrieved from each cow. A total of three swabs of vaginal content were collected per cow. One swab was collected for 16S rRNA sequencing, placed in a sterile tube, and transported on ice to the laboratory. At the laboratory, the swab was placed in 2 mL sterile microtubes and stored at −80 °C for further processing. Two swabs were collected for cfu counts, placed into tubes containing specific transport media (Versa TREK REDOX 1 and Versa TREK REDOX 2, Thermo Fisher Scientific), and transported to the laboratory on ice where they were placed in conical centrifuge tubes containing 2 mL of the specific culture medium (same used for transportation) and processed according to the methods previously described.

### 2.9. Metritis Definition

The evaluation of vaginal discharge was performed daily by two research members blinded to the challenge assignment. For the purpose of this study, cows presenting puerperal metritis or clinical metritis were classified as metritic cows. According to the characteristics of content retrieved using the Metricheck device, the vaginal discharge score (VDS) varied from 0 to 3: 0 (clear lochia with viscous discharge or no discharge observed), 1 (clear mucus with <50% of purulent of mucopurulent content), 2 (clear mucus with ≥50% of purulent or mucopurulent content), and 3 (fetid, watery, red-brownish uterine discharge disregarding of fever). Cows presenting VDS equal to 3 were considered with metritis. Although the scheme used for the diagnosis of metritis is validated for endometritis, it was used to evaluate the progression of the VDS until the development of metritis. In spite of that, the case definition of metritis (VDS equals to 3) complies with the classification proposed by Sheldon, Lewis [7].

### 2.10. DNA Extraction and Microbiome

The DNA extraction was carried by adding 1 mL of UltraPure^TM^ distilled water (DNAse and RNAse free, Invitrogen; Grand Island, NY, USA) into the conical centrifuge tube containing the swab followed by 10 min of vortex homogenization (Fisher Scientific; Hampton, NH, USA). Swabs were discarded and the liquid was centrifuged in room temperature for 5 min at 16,200× *g*. The pellet was submitted to DNA extraction using the DNeasy PowerFood Microbial Kit (Qiagen; Hilden, Germany) according to the manufacturer’s instructions.

To perform DNA sequencing, a PCR was carried using primers 515F and 806R for amplification of the V4 hypervariable region of the bacterial/archaeal 16S rDNA gene following the method previously optimized for the Illumina MiSeq platform [32]. Samples of the extracted DNA were amplified using different barcodes for 16S rRNA gene PCR with a 12 bp error-correcting Golay (http://www.earthmicrobiome.org, accessed on 25 November 2020). For PCR, 10 μM of EconoTaq Plus Green 1× Master Mix (Lucigen^®^; Middleton, WI, USA), 10 ng–100 ng of DNA, and UltraPure^TM^ distilled water (DNAse and RNAse free, Invitrogen) was used to achieve the final volume of a 50 μL reaction. The PCR steps were: 94 °C for 3 min for initial denaturing; 35 cycles of 45 s at 94 °C, 1 min at 50 °C, 90 s at 72 °C; and 10 min at 72 °C for the final elongation. For verification of amplicon presence, the amplified DNA was loaded in agarose gel (1.2%, wt/vol) containing 0.5 mg/mL of ethidium bromide. Amplified DNA was purified using the Mag-Bind^®^ Total Pure NGS (Omega Bio-Tek Inc.; Norcross, GA, USA) following the manufacturer’s instructions. The spectrophotometric estimation was used (A_260_ of 1.0 = 50 µg/mL pure dsDNA) to determine the DNA concentration. The quantified DNA was diluted to similar concentrations and pooled to prepare the library, then sequenced with the MiSeq platform (Illumina Inc.; San Diego, CA, USA) using Reagent MiSeq V2 300 cycles. The PCR, purification, and pooling procedures were automized using the OT-2 robot pipetting (Opentrons; New York, NY, USA).

After sequencing, the MiSeq Reporter Metagenomics Workflow was used to generate the Operational Taxonomic Unit (OTU) tables based on the Greengenes database (http://greengenes.lbl.gov/, accessed on 25 November 2020). The classification of reads in the output used from this workflow has a classification of reads in multiple levels, where we analyzed the phylum and genus.

### 2.11. Data Analysis

Analysis of descriptive statistics was performed using SAS (SAS Institute Inc.). Continuous data collected over time (e.g., RT, milk production, cfu count, 16S rRNA sequences, plasma acute phase proteins and cytokines, and serum metabolites) was analyzed using general linear mixed models via the PROC MIXED procedure of SAS version 9.4 (SAS Institute Inc.). Residual plots were used to assess the normality and homoscedasticity of residuals. Models included the fixed effects of treatment (control, low-dose, and high-dose), day after challenge and the interaction term between treatment and days after challenge, and random effects included the block and animal within block. As some continuous data did not follow the normal distribution, the results were transformed to log10 (cfu counts and relative abundance of some genus) or to the natural logarithm (plasma concentration of SAA and haptoglobin, and the relative abundance of the genus *Escherichia*). Based on the Akaike information criterion, the covariance structure with the lowest AIC was selected for each dependent variable.

Dichotomous outcomes such as the incidence of metritis and fever were evaluated using multivariate logistic regression models and the binary distribution of the GLIMMIX procedure (SAS version 9.4). The model included the random effect of the block and the fixed effect of treatment.

For all models, variables were considered statistically significant when a *p*-value ≤ 0.05 was detected, and a tendency to significance was considered if the *p*-value was between 0.05 and 0.10. In all models, Fisher’s Protected LSD was used for multiple comparisons such that pairwise treatment comparisons were performed only if the treatment effect or treatment by days after the challenge effect was significant at the 0.05 level.

## 3. Results

### 3.1. Descriptive Characteristics, Metritis Incidence, Rectal Temperature, Dry Matter Intake, and Milk Production

A detailed analysis for BCS, BW, and RT at enrollment; age at calving in days; days of gestation; and genomic tests was performed, revealing no differences among groups (Table 1). The average time in h from parturition to the challenge for each group was similar. The control cows were infused with the sterile saline solution in an average interval of 12.1 h, ranging from 6.0 to 19.3 h (SD = 4.8 h), while the low-dose and high-dose cows were challenged with a mean interval of 12.4 h, ranging from 6.2 to 18.3 h (SD = 4.37 h), and 14.2 h, ranging from 9.9 to 19.7 h (SD = 3.5 h), respectively.

**Figure 1:**
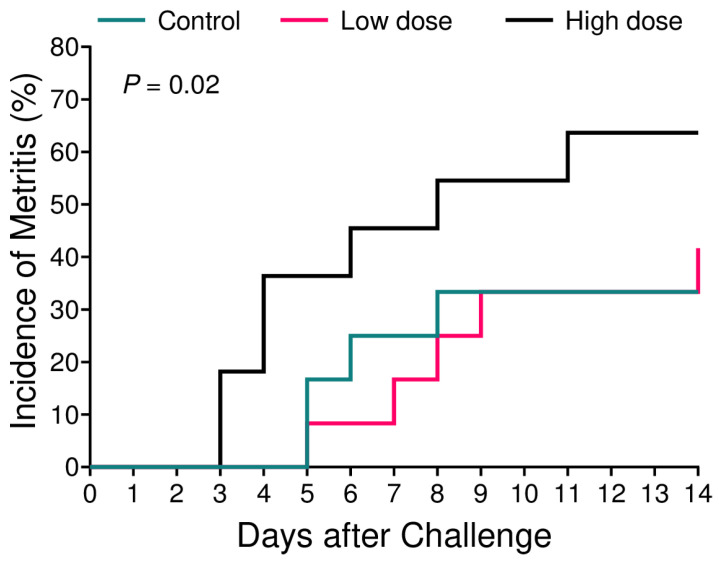
Kaplan–Meier survival curves for calving to metritis diagnosis of cows challenged intrauterine with a bacterial inoculum containing 10^3^ cfu (*n* = 12) or 10^6^ cfu (*n* = 11) of *E. coli*, *T. pyogenes*, and *F. necrophorum*, and controls (*n* = 12).

There was no difference in the metritis incidence among groups over the course of the study. The controls had a 33% (4/12) incidence of metritis, whereas the low-dose and high-dose groups had a 42% (5/12) and 64% (7/11) incidence (control vs low-dose: *p* = 0.67; control vs high-dose: *p* = 0.16), respectively. However, survival analysis revealed a difference (*p* = 0.02) among groups in the hazard of being diagnosed with metritis. The high-dose group had a 2.7 times greater hazard of being diagnosed with metritis when compared with the controls (*p* = 0.02; high-dose vs control hazard ratio = 2.7, 95% confidence interval = 1 to 6.5), with this difference starting on study d 3, while the low-dose group had a similar risk of being diagnosed with metritis compared with the control cows (*p* = 0.80; low-dose vs control hazard ratio = 1.2, 95% confidence interval = 0.3 to 4.8). The data are presented in Figure 1.

**Figure 2:**
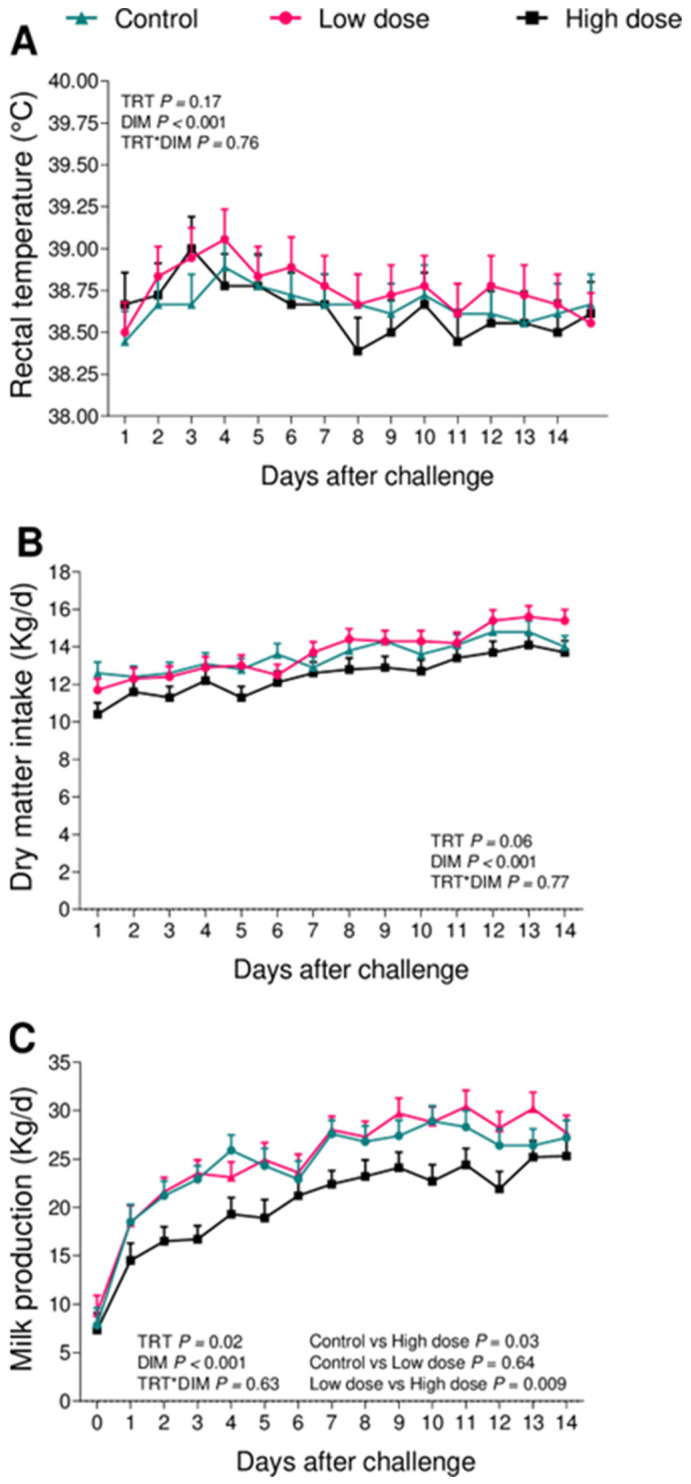
Clinical signs during the study period. Rectal temperature (**A**), milk production (**B**), and dry matter intake (**C**) during the first 14 days of lactation of cows challenged intrauterine with a bacterial inoculum containing 10^3^ cfu (*n* = 12) or 10^6^ cfu (*n* = 11) of *E. coli*, *T. pyogenes*, and *F. necrophorum*, and controls (*n* = 12). Results are presented as LSM ± SEM. TRT = treatment.

The bacterial challenge did not affect RT (*p* = 0.17; Figure 2A). When analyzing the fever incidence, the control cows had a lower incidence of fever when compared with the low-dose cows (control = 8.3% vs. low-dose = 50.0%; *p* = 0.06), but no difference when compared to the high-dose cows (control = 8.3% vs. high-dose = 36.4%; *p* = 0.15).

The DMI tended to be reduced (*p* = 0.06) by the bacterial challenge (Figure 2B). Overall, the high-dose cows consumed on average 1.1 (*p* = 0.06) and 1.2 (*p* = 0.03) kg/d less dry matter when compared with the control and low-dose cows, respectively, while the control and low-dose animals were not different (*p* = 0.73).

Milk production was affected by the bacterial challenge (*p* = 0.02; Figure 2C). Overall, the high-dose group produced an average of 20.2 ± 1.2 kg/d of milk, while the control and low-dose produced 24.2 ± 1.2 and 25.0 ± 1.2 kg/d, respectively. The high-dose group produced on average 3.9 (*p* = 0.03) and 4.7 (*p* = 0.009) kg/d less milk when compared to the control or low-dose groups, whereas no differences were observed when comparing the control and low-dose groups (*p* = 0.64). There were no differences in BCS (*p* = 0.64) and BW (*p* = 0.56) loss between the challenge groups during the first 14 days in milk.

### 3.2. Plasma Concentration of Acute Phase Proteins and Cytokines, Serum Concentration of Metabolites, and Complete Blood Cell Count

There were no differences in the plasma concentration of SAA, haptoglobin, and LBP (SAA: *p* = 0.26, SAA back transformed: *p* = 0.29, haptoglobin: *p* = 0.31, haptoglobin back transformed: *p* = 0.30, LBP: *p* = 0.24) (Figure S1).

**Figure 3:**
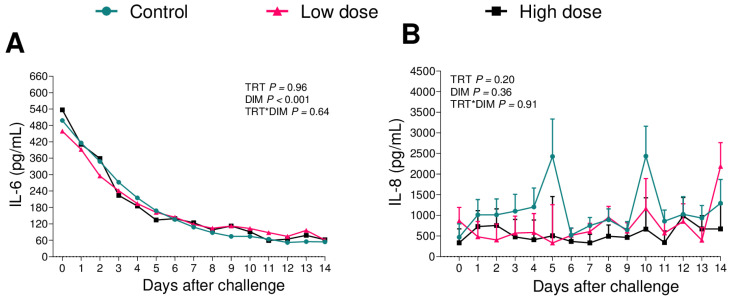
Plasma concentration of IL-6 (**A**) and IL-8 (**B**) during the first 14 d of lactation of cows challenged with low-dose (*n* = 12) and high-dose (*n* = 11) of bacterial inoculum or controls (*n* = 12). Results are presented as LSM ± SEM. TRT = treatment.

We measured the plasma concentrations of the pro-inflammatory cytokine TNFα, IL-2, and IL-6; the chemokine IL-8; and the anti-inflammatory IL-10. The TNF-α, IL-2, and IL-10 were below the limit of detection for the commercial ELISA assays used. The detectable cytokines were IL-6 and IL-8 (Figure 3), and these were not affected by the challenge administration (IL-6: *p* = 0.96, IL-8: *p* = 0.20).

There were no differences in the calcium serum concentration among groups (*p* = 0.58), but an interaction of the challenge and time (*p* = 0.02) was detected (Figure S2). The bacterial challenge did not affect the serum FA (*p* = 0.67), BHB (*p* = 0.40), albumin (*p* = 0.42), total protein (*p* = 0.87), or glucose (*p* = 0.19) concentrations (Figure S2). Liver damage was assessed by measuring the serum levels of lactate (*p* = 0.28) and the hepatic proteins AST (*p* = 0.12), ALT (*p* = 0.43), ALP (*p* = 0.44), GGT (*p* = 0.68). None of these were affected by the challenge (Figure S3). When analyzing the complete blood cell count, there were no differences in the numbers of white blood cells (*p* = 0.40), lymphocytes (*p* = 0.99), monocytes (*p* = 0.38), or granulocytes (*p* = 0.25; Figure S4).

### 3.3. Vaginal Microbiology

**Figure 4:**
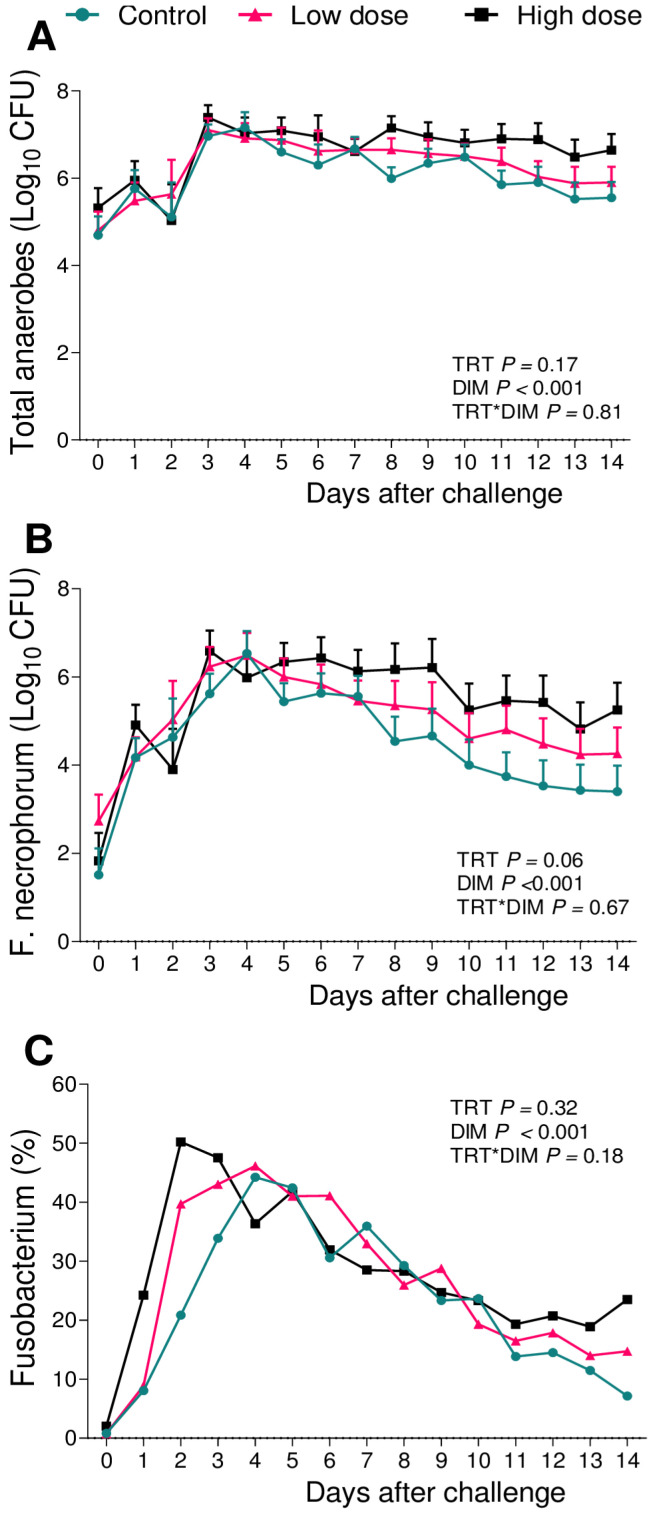
Bacterial cultures of total anaerobes (**A**) and *F. necrophorum* (**B**), and relative abundance of the genus *Fusobacterium* (**C**) from vaginal content during the first 14 d of lactation of cows challenged intrauterine with a bacterial inoculum containing 10^3^ cfu (*n* = 12) or 10^6^ cfu (*n* = 11) of *E. coli*, *T. pyogenes*, and *F. necrophorum*, and controls (*n* = 12). Results are presented as LSM ± SEM. TRT = treatment.

The bacterial challenge tended to alter the bacterial counts for *F. necrophorum* (*p* = 0.06; Figure 4B), but with no effect of the challenge on the total anaerobes’ cfu (*p* = 0.17; Figure 4A). Unexpectedly, the relative abundance of 16S sequence reads of the genus *Fusobacterium* was not altered (*p* = 0.32; Figure 4C).

**Figure 5:**
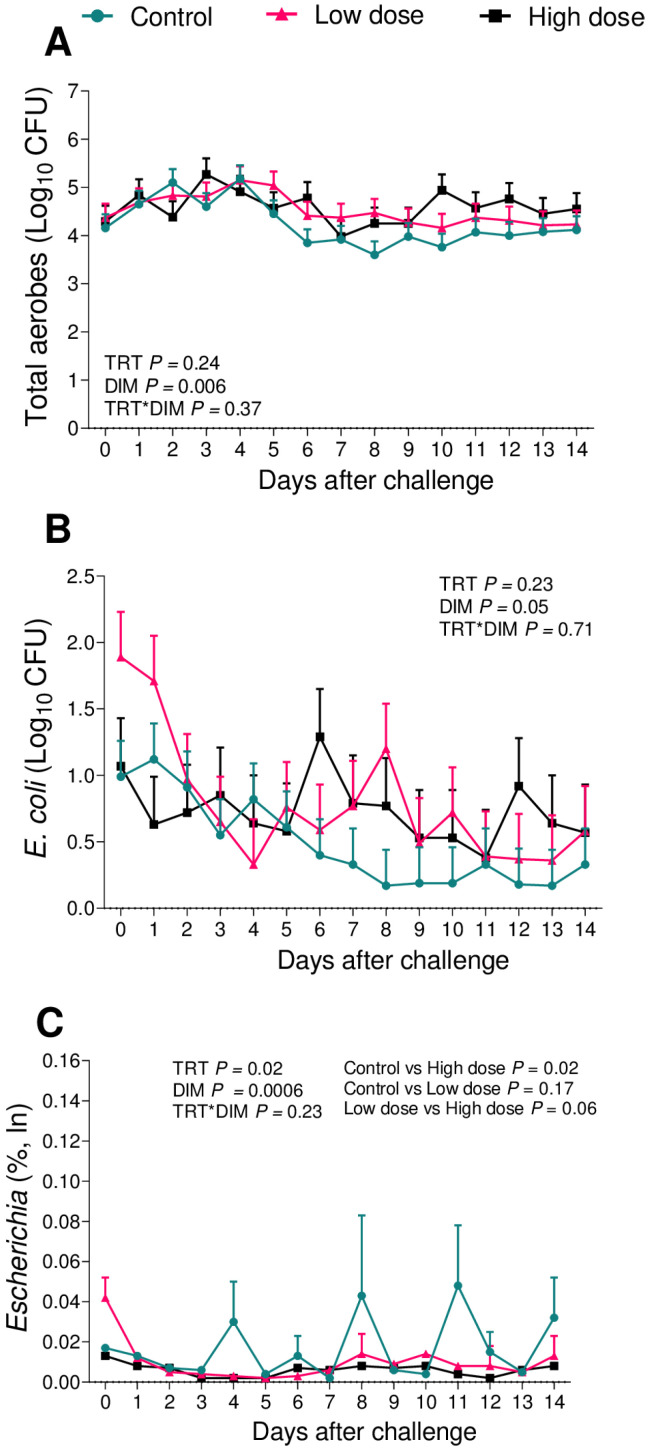
Bacterial cultures of total aerobes (**A**) and *E. coli* (**B**), and relative abundance of the genus *Escherichia* (**C**) from vaginal swabs during the first 14 d of lactation of cows challenged intrauterine with a bacterial inoculum containing 10^3^ cfu (*n* = 12) or 10^6^ cfu (*n* = 11) of *E. coli*, *T. pyogenes*, and *F. necrophorum*, and controls (*n* = 12). ln = natural log. Results are presented as LSM ± SEM. TRT = treatment.

**Figure 6:**
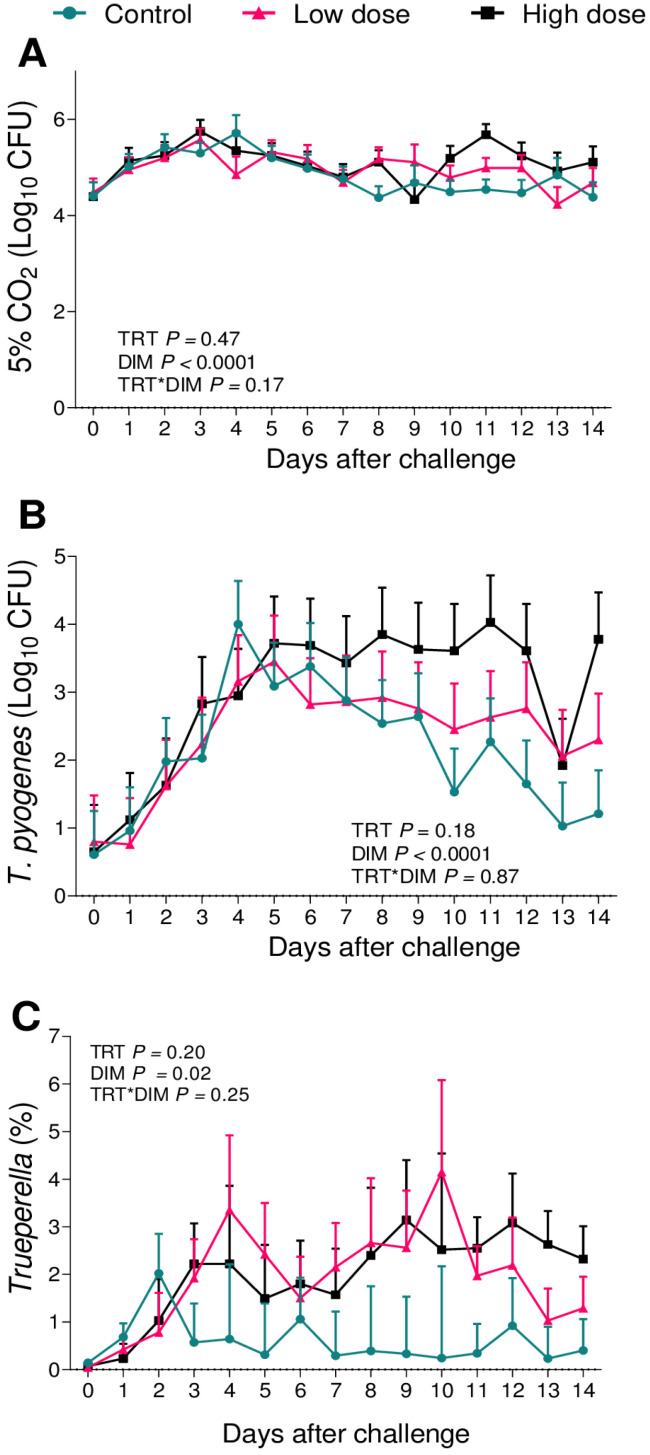
Bacterial cultures of total facultative anaerobes (5% CO_2_; (**A**)) and *T. pyogenes* (**B**), and relative abundance of the genus *Trueperella* (**C**) from vaginal content during the first 14 d of lactation of cows challenged intrauterine with a bacterial inoculum containing 10^3^ cfu (*n* = 12) or 10^6^ cfu (*n* = 11) of *E. coli*, *T. pyogenes*, and *F. necrophorum*, and controls (*n* = 12). Results are presented as LSM ± SEM. TRT = treatment.

There were no differences in the cfu of the total aerobes (*p* = 0.24; Figure 5A) or *E. coli* (*p* = 0.23; Figure 5B). There was an effect of the challenge on the relative abundance of 16S sequence reads of the genus *Escherichia* (*p* = 0.02; Figure 5C). High-dose cows presented a greater relative abundance of 16S sequence reads of the genus *Escherichia* when compared to the controls (*p* = 0.02)a and a tendency to a greater relative abundance when compared to the low-dose (*p* = 0.06). There was no effect of the challenge on the cfu of the total facultative anaerobes (5% CO_2_; *p* = 0.47; Figure 6A), *T. pyogenes* (*p* = 0.18; Figure 6B), or the relative abundance of 16S sequence reads of the genus *Trueperella* (*p* = 0.20; Figure 6C) among groups.

**Figure 7:**
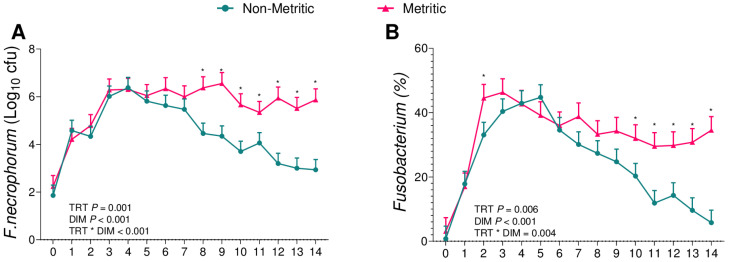
Bacterial cultures of *F. necrophorum* (**A**) and relative abundance of the genus *Fusobacterium* (**B**) from vaginal content during the first 14 d of lactation of cows diagnosed with metritis (*n* = 16) and cows diagnosed as healthy (*n* = 19). * *p* = 0.05 (non-metritic vs. metritic). Results are presented as LSM ± SEM. TRT = treatment.

**Figure 8:**
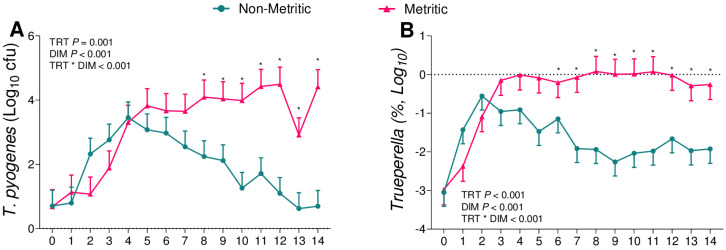
Bacterial cultures of *T. pyogenes* (**A**) and relative abundance of the genus *Trueperella* (**B**) from vaginal content during the first 14 d of lactation of cows diagnosed with metritis (*n* = 16) and cows diagnosed as healthy (*n* = 19). * *p* = 0.05 (non-metritic vs. metritic). Results are presented as LSM ± SEM. TRT = treatment.

**Figure 9:**
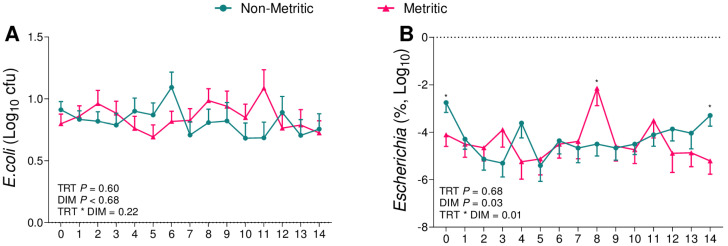
Bacterial cultures of *E. coli* (**A**) and relative abundance of the genus *Escherichia* (**B**) from vaginal swabs during the first 14 d of lactation of cows diagnosed with metritis (*n* = 16) and cows diagnosed as healthy (*n* = 19). * *p* = 0.05 (non-metritic vs. metritic). Results are presented as LSM ± SEM. TRT = treatment.

We then further compared cows diagnosed with metritis, independently of the challenge assigned, to cows not diagnosed with metritis. Metritic cows had greater bacterial counts of *F. necrophorum* (*p* = 0.001; Figure 7A) and *T. pyogenes* (*p* = 0.001; Figure 8A) and a greater relative abundance of 16S reads of the genera *Fusobacterium* (*p* = 0.006; Figure 7B) and *Trueperella* (*p* < 0.001; Figure 8B). On the other hand, there was no difference between both groups for the bacterial count for *E. coli* (*p* = 0.60; Figure 9A) or for the relative abundance of 16S sequence reads of the genus *Escherichia* (*p* = 0.68; Figure 9B).

## 4. Discussion

Our group has recently developed a novel metritis challenge model in postpartum multiparous dairy cows using an intrauterine inoculation of a bacterial cocktail containing either 10^6^ cfu or 10^9^ cfu of *E. coli*, *T. pyogenes*, and *F. necrophorum*. The model successfully induced clinical signs associated with metritis in 83% of cows receiving the lower dose, which is 10^6^ cfu of each bacterial species. Moreover, the same group had lesser DMI, milk production, and a greater concentration of systemic inflammation biomarkers [25]. Intriguingly, cows receiving 10^9^ cfu of the same challenge did not increase the incidence of metritis. Based on these findings, we designed a metritis induction model for primiparous cows using the same combination of microorganisms (*E. coli*, *T. pyogenes*, and *F. necrophorum*) in two different doses, 10^3^ and 10^6^ cfu, within 24 h postpartum. In primiparous cows, the challenge administration did not increase the incidence of metritis among groups. Nevertheless, cows challenged with 10^6^ cfu (high-dose group) had a greater hazard of being diagnosed with metritis when compared with the controls (Figure 1) and presented clinical signs such as lesser DMI (Figure 2B) and reduced milk production (Figure 2C). Also, the high-dose group had a greater growth of *F. necrophorum* (Figure 4B).

We expected the activation of the immune system with the administration of the challenge to postpartum cows, increasing the plasma concentration of chemokines, including IL-8 and pro-inflammatory cytokines such as TNF-α, IL-6, and IL-2 [33,34]. Intriguingly, our findings seem to contradict this theory, as we were unable to detect TNF-α, IL-2, and IL-10, and the challenge administration did not affect IL-6 or IL-8 (Figure 3). Although there was evidence of an acute response, there was no effect of the challenge on the measured acute phase proteins (Figure S1). According to Sheldon, Molinari [35], although most cows are exposed to pathogens during parturition, those who were able to employ strategies such as avoidance, tolerance, and resistance became resilient animals and remained healthy. According to the author, avoidance is the result of an intrinsic behavior, with cows naturally avoiding pathogens, reducing the risk of diseases; tolerance is the ability of cows to limit the damage caused by bacteria; and resistance is a mechanism that limits infection via pathogenic bacteria, activating the innate and adaptative immune system. Based on this, we hypothesize that primiparous are able to tolerate and resist bacterial infection in a greater way when compared to multiparous cows.

The innate immune response plays a role in protecting the uterus of postpartum cows, and it has been widely described [6,36]. During the postpartum period, multiparous cows usually experience a negative energy balance (NEB) as feed intake is not sufficient to meet the demands for growth, maintenance, and milk production [3,37]. During NEB, polymorphonuclear (PMN) activity is impaired, as their phagocytic and killing capacities are reduced [38,39], favoring the development of diseases such as metritis. Primiparous cows experience a less pronounced NEB [23] and a less severe postpartum neutrophil impairment [38]. We observed that the group challenged with the higher dose of the bacterial cocktail had an increased incidence of disease, but when compared with the controls, this difference was not significant, suggesting that the innate immune system in primiparous cows was more effective to combat invading microorganisms and prevent disease.

Although the role of adaptative immunity in preventing diseases after parturition is not yet understood, aggregates of B and T-lymphocytes were found via the endometrium of postpartum cows [40,41]. Also, Dhaliwal, Murray [42] reported that the uterine fluid of postpartum cows contains IgA and IgG, confirming the protective role of serum immunoglobulins in the uterus. Based on this, we observed that after the parturition, all groups had an increase in *F. necrophorum* (Figure 4B) and *T. pyogenes* (Figure 6B) that was later reduced in cows that remained healthy (Figure 7 and Figure 8). We therefore hypothesize that as primiparous cows were not previously exposed to these pathogens, it took time to mount an immune response and control the growth of pathogenic bacteria, but the immune system was very effective in controlling bacterial growth once activated.

When associating the data obtained from the uterine microbiome and the bacterial culture, we observed that among the infused microorganisms, *T. pyogenes* (Figure 6) and *E. coli* (Figure 5) were not the main pathogens for the development of metritis, as previously described [26,28,29,43]. In the case of this challenge model, *F. necrophorum* (Figure 4) tended to affect the development of disease in cows receiving 10^6^ cfu of the challenge. Furthermore, when we compared the uterine microbiome of cows that developed metritis with cows that remained healthy during the study period, we observed that *F. necrophorum* (Figure 7) and *T. pyogenes* (Figure 8) play a role in the development of metritis, while the same is not observed for *E. coli* (Figure 9). We therefore suggest that *F. necrophorum* may play a role in the development of metritis.

This study demonstrated the importance of genomic traits to predict the development of diseases such as metritis, as demonstrated in a recent study [44]. In that study, cows were ranked in quartiles based on the standardized transmitting ability to develop a disease (Table 1). Referring to metritis, animals from the bottom 25th percentile had an incidence of metritis of 23.64%, whereas animals from the top 25th percentile had an incidence of metritis of 12.86%. These results highlight the role of genetics in predisposing an animal to develop a disease; in this example, it is clinical metritis.

The study also brings awareness about the importance regarding the cleaning of facilities at the maternity during parturition, and the good hygiene of employees when providing assistance to a difficult parturition. The control cows were not submitted to a challenge and were able to overcome the postpartum period without the development of diseases.

Although the present study was carefully designed to control for confounders, we are aware that our research has limitations that deserve a detailed discussion. We associated the data obtained for bacterial growth with 16S rRNA gene sequencing to describe the dynamics of microorganisms from the challenge to the development of metritis. We observed a 1.5-fold difference (*p* = 0.06) on *F. necrophorum* growth on the selective media (LKV agar plates) for the high-dose group compared with the controls (Figure 4B), but we did not observe an effect of the challenge when analyzing the relative abundance of the genus *Fusobacterium* when using 16S rRNA sequencing (Figure 4C). This could be related to the different specificity of each measurement. The 16S rRNA sequencing data was analyzed considering the relative abundance of each genus within the bacterial population of the uterus, while in the bacterial culture, we counted the growth of each microorganism in a selective medium; therefore, the difference observed could be attributed to it. We also acknowledge that it would be ideal to infuse the control cows with the culture medium instead of using the sterile saline solution, but we used the sterile saline solution to prevent the occasional inoculation of any possible bacterial contamination to the uterus.

Another point of discussion for this study is the fact that multiparous cows challenged with 10^9^ cfu of the bacterial challenge had a similar incidence of metritis when compared with cows that were infused with the sterile saline solution [25]. We also considered the uterus of primiparous could be smaller when compared to the uterus of multiparous cows [45]. Therefore, we used a lower dose of bacteria (10^3^ cfu) than we did in our previous model [25]. Unfortunately, we observed that inoculating 10^3^ cfu was not efficacious for inducing metritis in primiparous cows, and we may consider in the future the use of a higher dose, such as 10^9^ cfu, to be able to overwhelm the immune system of these cows, as these cows did not contract metritis to the same extent as the older cows.

The present study was designed to follow up on the successful metritis induction model for multiparous cows, in which we enrolled three groups of 12 cows, producing a significant difference in metritis induction when comparing the 10^6^ dose with the controls [25]. Based on the results of that trial, we considered that the same result could be obtained when inducing metritis in primiparous cows after controlling for predisposing factors for the development of metritis. Unfortunately, the current study design lacked the power to detect differences between the high-dose group and the controls because we expected a greater effect of the challenge in inducing metritis. The ideal experiment would be to enroll 23 primiparous cows per group to generate a significant difference based on the incidence of metritis observed in the control cows.

With the present report, we propose a change in the way that the analysis of microbiome data is performed. With our model of metritis induction in multiparous cows [25], we observed that the genus *Fusobacterium* was the most relevant for the development of metritis. We also observed that primiparous cows that developed disease had a greater abundance of this genus when compared to healthy cows, although the challenge dose used was not able to increase the cases of metritis in challenged cows when compared to the controls. From this finding, we speculate that the dynamics of microorganisms for the development of metritis in primiparous cows are different than those observed in multiparous cows. The reports that discuss the microbiome in a metritic uterus do not stratify or compare primiparous and multiparous cows, as the focus is on the comparison of microorganisms present in the uterus of metritic and non-metritic cows [12,46,47]. We therefore suggest that further research could focus on the dynamics of microorganism growth for the development of metritis, stratifying primiparous and multiparous cows. We also propose that further research should be conducted to better understand the differences in metritis induction between primiparous and multiparous cows, and to better understand the dynamics of microorganisms and physiological factors that can be associated with the development of the disease in cows with differing numbers of parturitions.

## 5. Conclusions

We observed that a model that successfully induced clinical signs associated with metritis in multiparous Holstein cows did not induce metritis to the same extent in primiparous cows but, when accounting for time, increased the hazard ratio of metritis induction to 2.7 when comparing cows challenged with the inoculum containing 10^6^ cfu of *E. coli*, *T. pyogenes*, and *F. necrophorum* (high-dose group) to the control cows that received an intrauterine infusion of the sterile saline solution. We observed that the model tended to reduce the DMI and significantly decreased the milk production in cows enrolled in the high-dose group, whereas we did not observe any differences in rectal temperature, plasma cytokines levels, serum metabolites, or hepatic enzymes. Further research needs to be conducted to elucidate the difference in metritis induction in multiparous versus primiparous cows.

## Figures and Tables

**Table 1:** Descriptive data (LSM ± SEM) of cows challenged with low-dose (*n* = 12) and high-dose (*n* = 11) of bacterial inoculum or controls (*n* = 12).

Item ^1^	Control	Low-Dose	High-Dose	*p*-Value ^2^
BCS at enrollment	3.5 ± 0.07	3.4 ± 0.07	3.5 ± 0.08	0.64
BW at enrollment, kg	574.0 ± 19.4	590.9 ± 19.4	586.5 ± 20.8	0.56
RT at enrollment, °C	38.4 ± 0.2	38.5 ± 0.2	38.6 ± 0.2	0.17
Age at calving, d	661.6 ± 8.2	667.7 ± 8.2	673.4 ± 8.2	0.53
Days of gestation	275.4 ± 1.2	276.2 ± 1.2	276.1 ± 1.2	0.87
GMILK	657.9 ± 149.2	720.6 ± 155.8	751.0 ± 149.2	0.90
Z_MET	100.4 ± 1.1	102.7 ± 1.1	102.8 ± 1.1	0.24
Z_RP	98.0 ± 1.3	101.0 ± 1.4	102.1 ± 1.3	0.09
DWP	284.6 ± 46.8	272.6 ± 48.8	400.4± 46.8	0.10
Net Merit	235.0 ± 35	216.0 ± 36.5	261.7± 35	0.67
TPI	2219.4 ± 43	2192.1 ± 44.9	2231.1 ± 43	0.82

^1^ RT, Rectal temperature; GMILK, Genomic Enhanced Predicted Transmitting Ability for milk yield (Zoetis); Z_MET, Genomic Standardized Transmitting Ability for metritis risk (Zoetis); Z_RP, Genomic Standardized Transmitting Ability for retained placenta (Zoetis); DWP, Dairy Wellness Profit (Zoetis); Net Merit, Net Merit index (Zoetis); TPI, Total Performance Index (Holstein Association, Brattleboro, VT, USA). ^2^ *p*-value indicates the overall group effect.

## Data Availability

Data are available upon request from the corresponding author.

## Supplementary Materials

The following supporting information can be downloaded at: https://www.mdpi.com/article/10.3390/ani13182852/s1, Table S1: Ingredients (dry matter basis) of postpartum diet; Table S2: Chemical composition (dry matter basis) of postpartum diet; Figure S1: Plasma concentration of haptoglobin (A, B; transformed and back-transformed), SAA (C, D; transformed and back-transformed), LBP (E), and during the first 14 d of lactation of cows challenged intrauterine with a bacterial inoculum containing 10^3^ cfu (*n* = 12) or 10^6^ cfu (*n* = 11) of *E. coli*, *T. pyogenes*, and *F. necrophorum*, and controls (*n* = 12); Figure S2: FA (A), BHB (B), albumin (C), total protein (D), glucose (E), and calcium (F) levels during the first 14 d of lactation of cows challenged intrauterine with a bacterial inoculum containing 10^3^ cfu (*n* = 12) or 10^6^ cfu (*n* = 11) of *E. coli*, *T. pyogenes*, and *F. necrophorum*, and placebo controls (*n* = 12). ^†^ 0.05 < *p* < 0.10 (High-Dose vs. Low-Dose); Figure S3: Lactate (A), ALT (B), AST (C), ALP (D), and GGT (D) levels during the first 14 d of lactation of cows challenged intrauterine with a bacterial inoculum containing 10^3^ cfu (*n* = 12) or 10^6^ cfu (*n* = 11) of *E. coli*, *T. pyogenes*, and *F. necrophorum*, and controls (*n* = 12). Figure S4: Concentration of white blood cells (A), lymphocytes (B), monocytes (C), and granulocytes (D) during the first 14 d of lactation of cows challenged intrauterine with a bacterial inoculum containing 10^3^ cfu (*n* = 12) or 10^6^ cfu (*n* = 11) of *E. coli*, *T. pyogenes*, and *F. necrophorum*, and controls (*n* = 12).
