# Supplementary material for: Testing the Induction of Metritis in Healthy Postpartum Primiparous Cows Challenged with a Cocktail of Bacteria
Source: Animals (Basel). 2023 Sep 8;13(18):2852. doi: 10.3390/ani13182852 (PMC10525343; doi:10.3390/ani13182852)
Supplement: Supplementary file 1 [file animals-13-02852-s001.zip › animals-2582738-supplementary.pdf]

Table S1. Ingredients (dry matter basis) of postpartum diet.

Ingredient	% of DM
Corn silage	42.10
Hay crop silage	14.79
Whey Blend	2.93
Amino Plus[1]	2.08
Soybean hulls[1]	1.37
Soybean Meal[1]	3.56
Corn Meal[1]	18.99
Blood meal[1]	1.36
Salt	0.43
Berga Fat F-100[2]	1.09
MFP[3]	0.05
Express Meal – Dumond[1]	4.91
Smartamine [4]	0.13
Corn Gluten Feed [1]	3.68
Energizer Gold	0.48
Limestone Ground	0.62
S Carb	0.65
SS Lact Min (1625 g/ton)	0.77

[1]Button Nutrition Inc. (Skaneateles, NY). [2] Pure vegetable fat fraction, INNOCHEMS CO., LTD (VIETNAM). [3]MFP; Novus International (St. Charles, MO). [4]DL-Met, physically protected with pH-sensitive coating; Adisseo (Antony, France).

Table S2. Chemical composition (dry matter basis) of postpartum diet.

Energy and chemical composition[1]	DM	Supply
NE_L, Mcal/kg	-	1.60
NDF, %	29.94	17.37
NFC, %	42.60	24.70
ADF, %	24.35	14.12
Starch, %	27.42	15.90
CP, %	15.67	9.09
Ca, %	0.73	0.42
P, %	0.37	0.21
K, %	1.43	0.83
Na, %	0.45	0.25
Mg, %	0.26	0.15
S, %	0.22	0.13
CL, %	0.56	0.32
Zn, ppm	49.14	1,292.945
Cu, ppm	12.71	334.40
Se, ppm	0.099	2.60
I, ppm	0.29	7.53

[1] Chemical composition analysis was performed by Button Nutrition Inc., Skaneateles, NY.

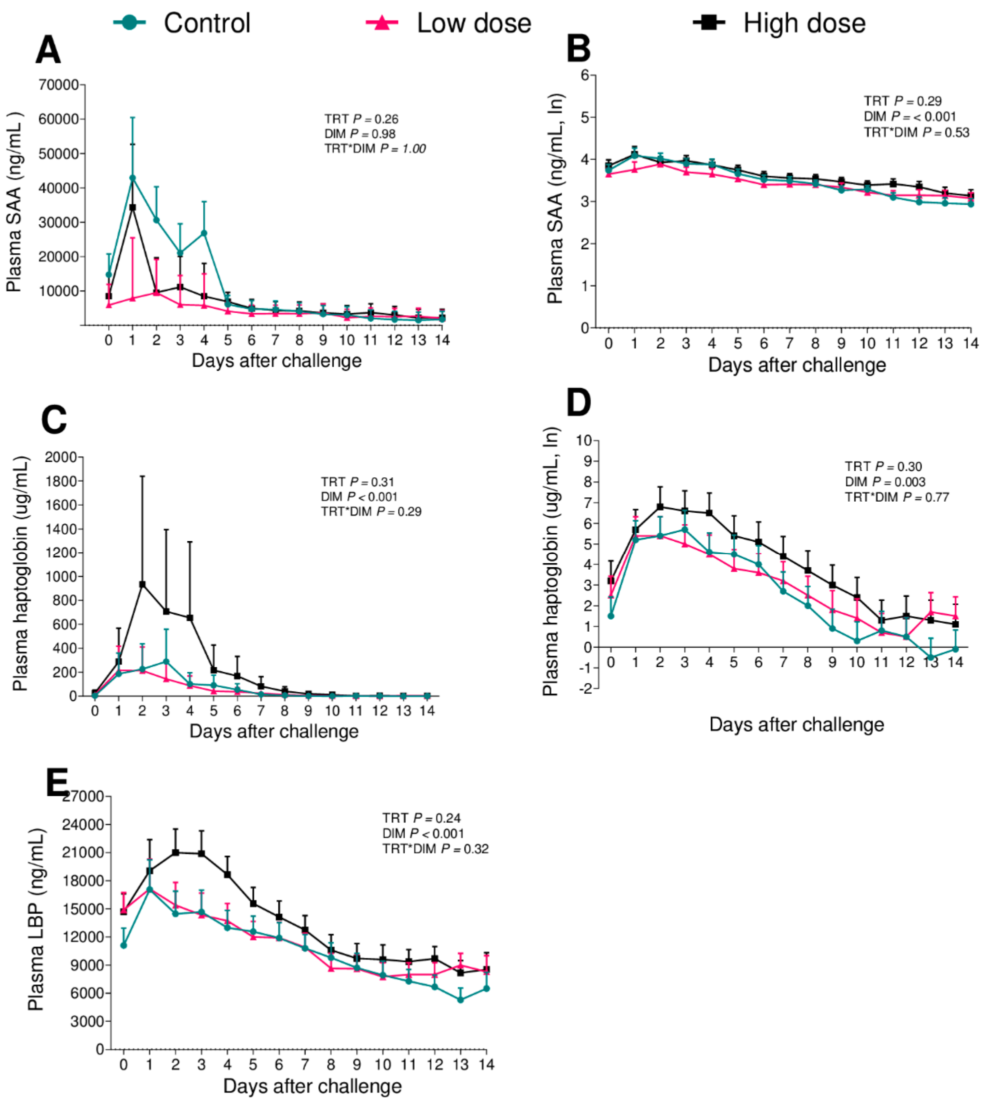

Figure S1. Plasma concentration of haptoglobin (A, B; transformed and back-transformed), SAA (C, D; transformed and back-transformed), LBP (E) and during the first 14 d of lactation of cows challenged intrauterine with a bacterial inoculum containing 10^3 cfu (n = 12), or 10^6 cfu (n = 11) of *E. coli*, *T. pyogenes*, and *F. necrophorum*, and controls (n = 12).

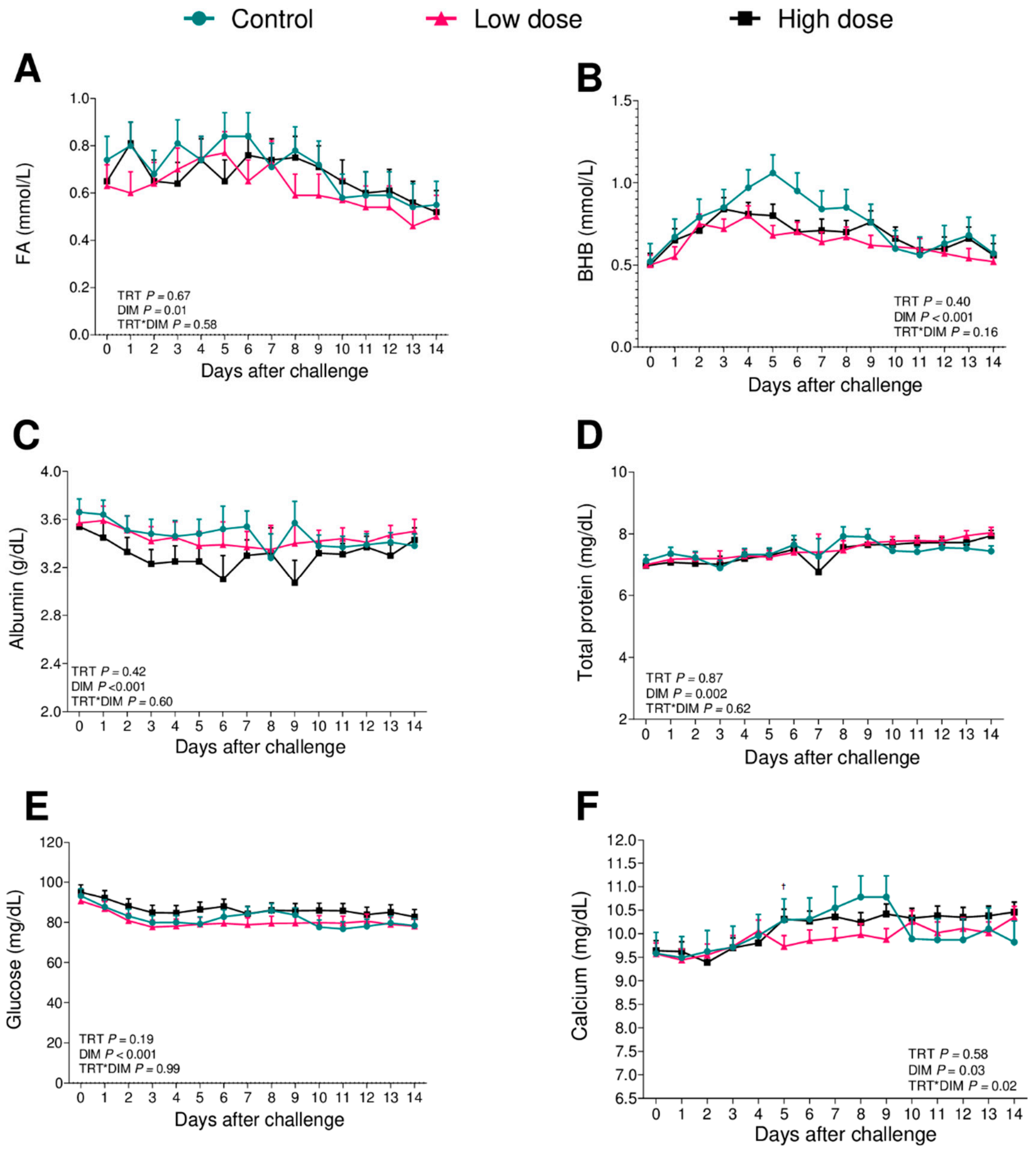

Figure S2. FA (A), BHB (B), albumin (C), total protein (D), glucose (E), and calcium (F) levels during the first 14 d of lactation of cows challenged intrauterine with a bacterial inoculum containing 10^3 cfu (n = 12), or 10^6 cfu (n = 11) of *E. coli*, *T. pyogenes*, and *F. necrophorum*, and placebo controls (n = 12). †0.05 < *P* < 0.10 (High-Dose vs Low-Dose).

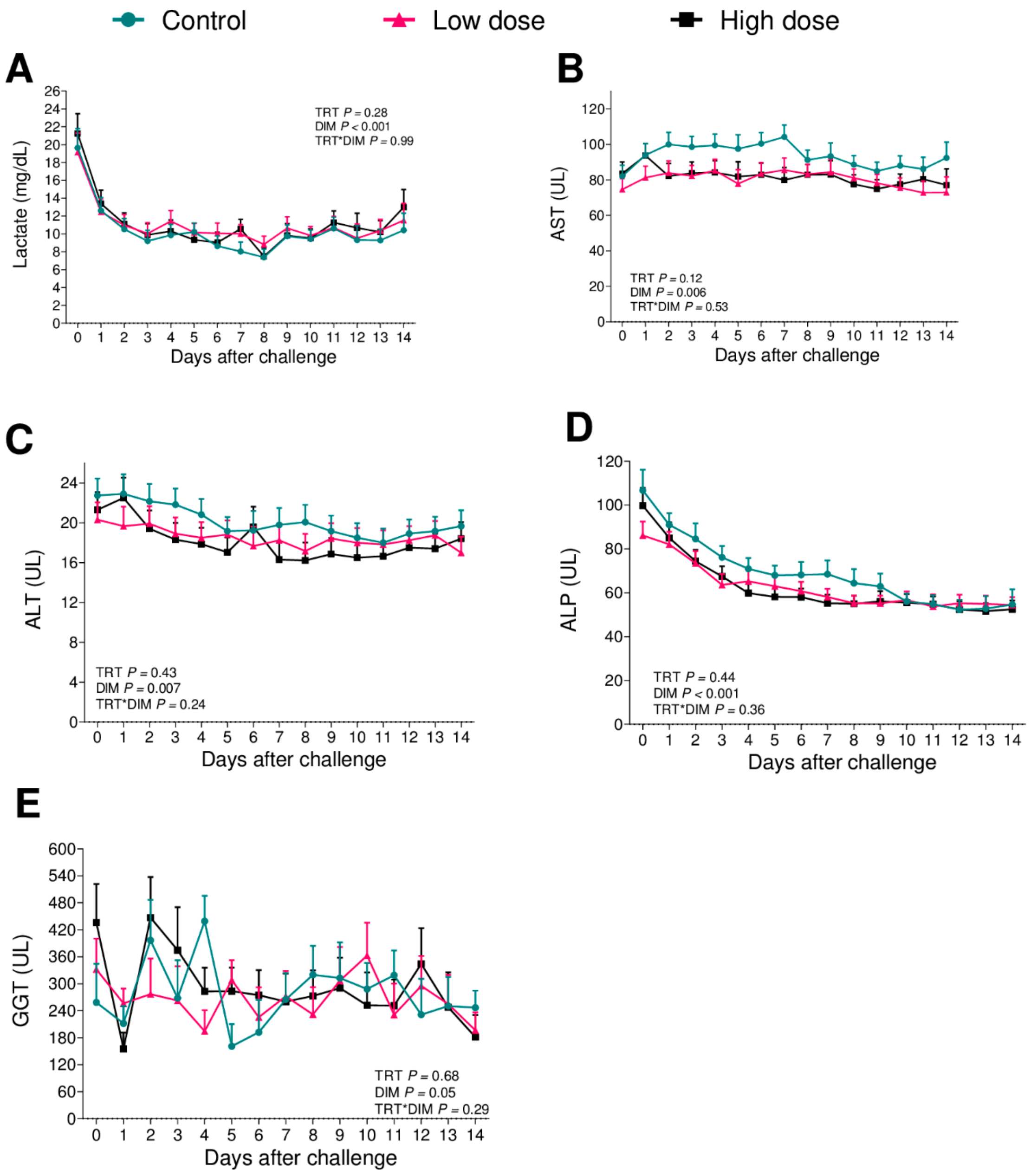

Figure S3. Lactate (A), ALT (B), AST (C), ALP (D), and GGT (D) levels during the first 14 d of lactation of cows challenged intrauterine with a bacterial inoculum containing 10^3 cfu (n = 12), or 10^6 cfu (n = 11) of *E. coli*, *T. pyogenes*, and *F. necrophorum*, and controls (n = 12).

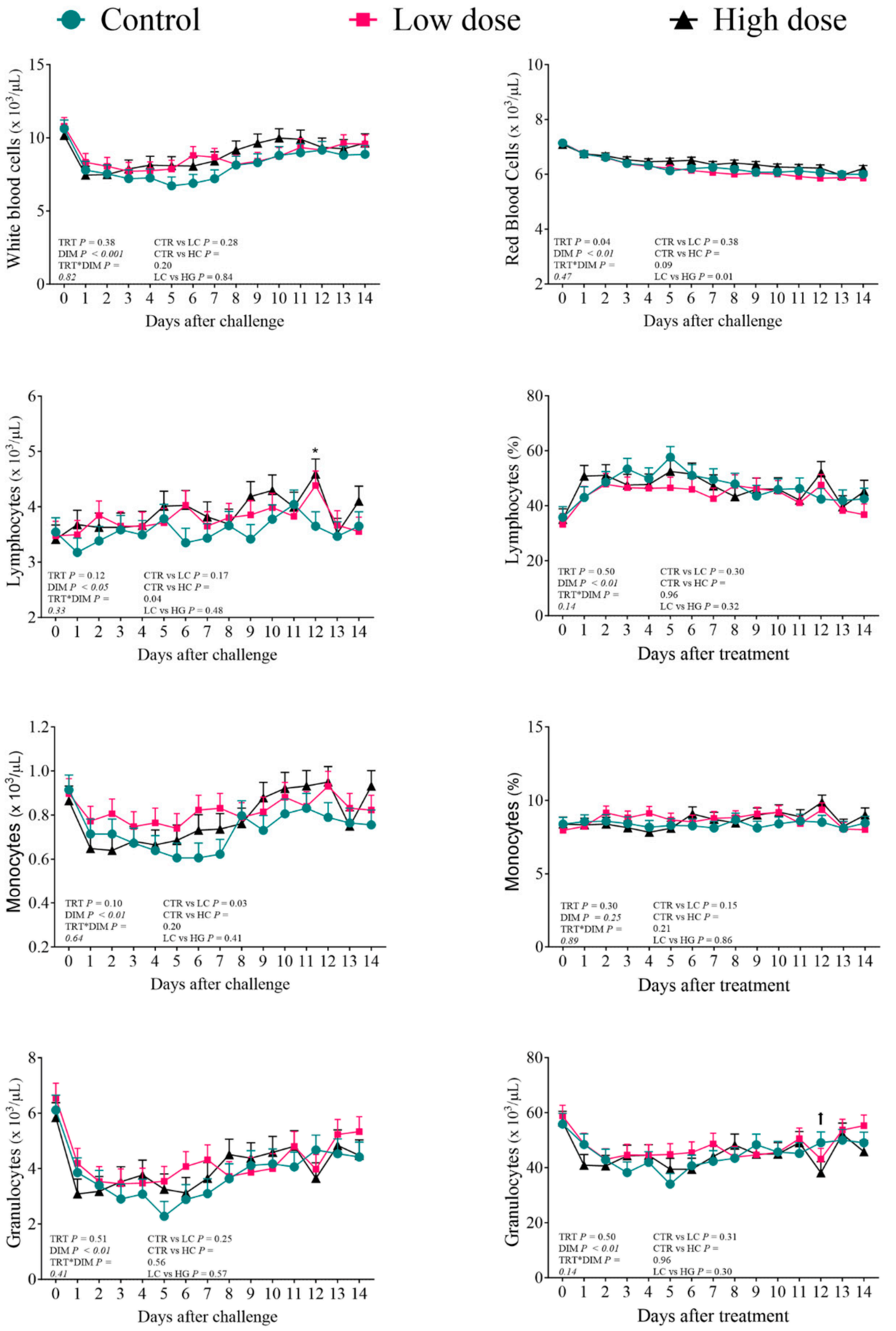

Figure S4. Concentration of white blood cells (A), lymphocytes (B), monocytes (C), granulocytes (D) during the first 14 d of lactation of cows challenged intrauterine with a bacterial inoculum containing 10^3 cfu (n = 12), or 10^6 cfu (n = 11) of *E. coli*, *T. pyogenes*, and *F. necrophorum*, and controls (n = 12).
